# Contrast-dependent modulation of gamma rhythm in v1: a network model

**DOI:** 10.1186/1471-2202-16-S1-O10

**Published:** 2015-12-18

**Authors:** Margarita Zachariou, Mark Roberts, Eric Lowet, Peter de Weerd, Avgis Hadjipapas

**Affiliations:** 1 University of Nicosia Medical School, Nicosia, Cyprus; 2 Psychology and Neuroscience, Maastricht University, Maastricht, the Netherlands; 3 Donders Institute for Brain, Cognition and Behaviour, Radboud University Nijmegen, Nijmegen, the Netherlands; 4 St. George's University of London, Cranmer Terrace, London, SW17 0RE, UK

## 

In our empirical data comprising of single-unit and LFP recordings in macaque area V1 and source reconstructed human MEG localized to visual cortex we have observed a robust increase in gamma oscillation frequency with increasing luminance contrast. In addition, at high grating contrasts, a robust decay in gamma power was observed in the LFP [1] but not the MEG. These phenomena are key to understanding the functional role of network frequencies and for investigating the stability of gamma oscillations at both local and macroscopic levels. However, even at the most basic level of spatially- undifferentiated neuronal models, it is not fully understood how excitatory (E) and inhibitory (I) neurons interact to generate the observed network gamma oscillations in the macaque single-unit and LFP data. For example we could obtain the frequency shift and power decay in a network where the rhythm is produced by excitatory neurons that fired more frequently than inhibitory neurons, and in another more neurophysiologically plausible network composed of excitatory neurons showing sparse firing [2,3] and inhibitory neurons showing faster firing [4]. Moreover, it is unknown how increasing excitatory afferent drive (of which luminance contrast is a proxy) modulates the interactions between E and I populations (as well as interactions within each population) to account for changes in frequency and power. We aimed to replicate the empirical data from macaque visual cortex and to further investigate the stability of the observed gamma oscillation. Here, we present an undifferentiated V1 network PING model, with realistic neuronal features as determined and validated from the analysis of a large number of V1 neurons obtained in 3 rhesus monkeys. The model when perturbed by increasing afferent input, exhibits the core characteristics of the empirical data, that is, (1) a monotonic increase in LFP frequency, (2) a non-monotonic LFP power modulation with decay at high inputs, (3) a largely non-saturating increase in average unit firing rate. In addition, the model exhibits realistic single unit behavior across a range of inputs. In terms of the frequency shift, we have observed remarkable scaling behaviour: while the frequency of oscillations changes dramatically with input, the absolute average phase at which inhibitory and excitatory neurons fire in each oscillation cycle and the average relative phase to each other remain constant. This scaling may on one hand underlie the stability of the gamma oscillation locally and on other hand facilitate communication through coherence in the gamma range [5] across varying stimulus conditions, by preserving the timing and relative ordering of population firing irrespective of the oscillation frequency [6]. Our results suggest that the observed power decline results from a primary (functional) decoupling among inhibitory neurons. Further analysis highlighted that the functional decoupling is related to the balance of inhibition/excitation. In further steps, we intend to test these predictions in the empirical data, and then proceed to a differentiated V1 columnar model to investigate the divergences between human MEG and macaque LFP/spiking responses.
